# Management of Super-refractory Postoperative Lumbar Cerebrospinal Fluid Leak: A Case Report and Review of the Literature

**DOI:** 10.7759/cureus.48215

**Published:** 2023-11-03

**Authors:** Phillip M Johansen, Payton Yerke Hansen, Denis Babici, Timothy D Miller

**Affiliations:** 1 Neurosurgery, University of South Florida, Tampa, USA; 2 Medicine, Florida Atlantic University Charles E. Schmidt College of Medicine, Boca Raton, USA; 3 Neurology, Marcus Neuroscience Institute, Boca Raton, USA; 4 Neurology, Florida Atlantic University Charles E. Schmidt College of Medicine, Boca Raton, USA; 5 Neurosurgery, Florida Atlantic University Charles E. Schmidt College of Medicine, Boca Raton, USA

**Keywords:** spine surgery, minimally invasive spinal surgery, hemilaminectomy, cerebrospinal fluid (csf), incidental dural tear

## Abstract

One well-documented risk of spinal surgery is cerebrospinal fluid (CSF) leak in the immediate postoperative period. While the majority of CSF leaks occur due to an obvious intraoperative dural tear, several reports have documented delayed CSF leakage from occult intraoperative dural tears. There is a paucity of published literature regarding the true incidence of dural tears in minimally invasive spinal surgery. Furthermore, the types of dural tears that require closure are poorly understood. According to the limited existing literature available, the recommended treatment of dural tears includes primary repair, subarachnoid drainage catheters, and blood patches. However, there are no distinct treatment guidelines between the different etiologies of CSF leakage. The most important aspect in the management of CSF leakage is prevention, including preoperative risk assessment and meticulous intraoperative manipulation. One emerging treatment strategy is to alter the pressure gradient in a manner that stops CSF leakage. This method is based on one of two mechanisms: direct suture or augmented closure with dural substitute material and either reducing the subarachnoid fluid pressure or increasing the epidural space pressure. Bed rest is a key element in the treatment of persistent CSF leaks, as it can reduce the lumbar CSF pressure, thereby preventing CSF leakage. We describe the challenging case of a persistent CSF leak despite multiple attempts at direct repair, as well as our management strategies. Understanding the proper positioning techniques to reduce leakage is crucial for proper management, and orthopedic surgeons, neurosurgeons, and neurointensivists may consider being more aggressive in treating persistent CSF leaks.

## Introduction

Cerebrospinal fluid (CSF) leakage is a dangerous complication of some spine surgeries because it can lead to meningitis, intracranial hypotension, impaired wound healing, and other complications. Current literature suggests that the incidence of intraoperative dural tears is somewhere between 0.5% and 18% [1]. While the majority of CSF leaks occur due to an obvious intraoperative dural tear, several reports have documented delayed CSF leakage from occult dural tears during surgery. Such cases present several days postoperatively with either drainage from the incision or symptoms of low CSF pressure such as headache, nausea and vomiting, diplopia, and gait unsteadiness. Oftentimes, physicians opt for a second operation to repair the dural tear [2]. Intraoperative dural tears can be repaired by primary closure if the tear is simple and easily repairable. One method that is frequently used in cases of large or laterally located dural tears is the utilization of a patch of deep fascia [3].

Management of persistent CSF leakage remains controversial. Many surgeons recommend for primary repair, while others advocate for a trial of CSF diversion with lumbar drainage [4]. Reoperation with direct dural closure is both invasive and may be technically challenging. The purpose of this article is to present our experience with a persistent postoperative CSF leak that was not recognized intraoperatively, as well as a unique solution to a particularly obstinate, morbidly obese patient, which made monitoring for CSF leakage especially challenging.

## Case presentation

A 31-year-old morbidly obese male with a past medical history of intervertebral disc herniation status post left L4-L5 hemilaminectomy and discectomy performed at another hospital three days prior presented to the emergency department with headache, radicular symptoms, and low back pain. The patient reported severe pain since his surgery, which had significantly limited his activity. His pain was initially localized to the lumbar spine and was more severe on the left than on the right side. He reported pain that radiated down the left leg and was refractory to analgesia.

**Figure 1:**
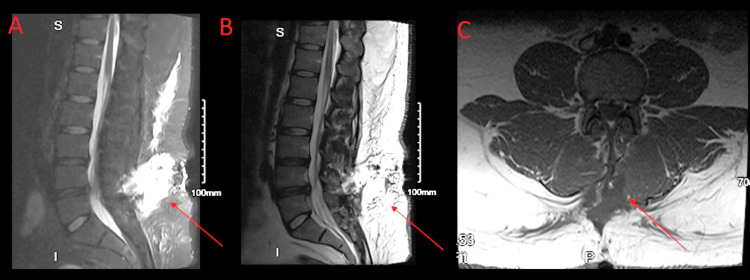
Postsurgical changes at L5-S1 with left hemilaminectomy A and B show a sagittal lumbar MRI, and C shows a coronal lumbar MRI. The arrows indicate fluid collection in the retrospinal soft tissues resulting in moderate narrowing of the thecal sac on the left, displacing the nerve roots toward the right within the canal. MRI: magnetic resonance imaging

Outside records indicated that the initial laminectomy procedure was complicated by an intraoperative CSF leak, which underwent primary repair. Postoperatively, there was no immediate evidence of active CSF leakage, and the patient was discharged home. However, over the course of the next day, he developed worsening pain and headaches, prompting him to return to the outpatient office of the orthopedic surgeon who performed his operation. At that time, his physical examination revealed a fluid collection at the wound site. The patient was sent back to the hospital for magnetic resonance imaging (MRI) of his lumbar spine, which was concerning for a dural spinal fluid leak along with a moderate, centrally extruded disk between L4 and L5 (Figure 1).

As a result, the orthopedic surgeon opted to return to the operating room for repair of the CSF leak in addition to a minimally invasive L4-L5 fusion.

**Figure 2:**
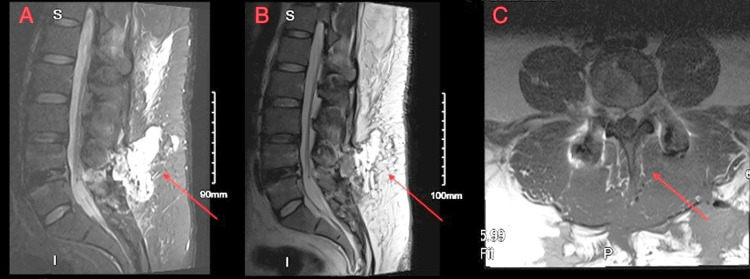
A and B show a sagittal lumbar MRI, and C shows a coronal lumbar MRI The arrows indicate persistent fluid collection in the retrospinal soft tissues resulting in moderate narrowing of the thecal sac on the left displacing the nerve roots toward the right within the canal. MRI: magnetic resonance imaging

The patient underwent a successful surgery and reported no pain or headaches for the first full day after surgery. However, after sitting up in bed for an extended period, his headache and lower extremity pain returned. In addition, the nursing staff noticed fluid leaking from his back. A repeat MRI of the lumbar spine showed a fluid collection posterior to the decompression extending through the paraspinal musculature all the way through the fascia. The decision was made to proceed with evacuation of the fluid collection, re-closure, and placement of a lumbar drain, followed by four days of bed rest limited to the supine position to allow for healing of the CSF leak and soft tissues. After the surgery, the lumbar drain yielded CSF at approximately 20 mL per hour. On postoperative day 4, the drain was removed by the orthopedic surgeon. Shortly after the lumbar drain was removed, the patient was found in the upright position, and there was evidence of repeat CSF leakage with saturation of several dressings. Subsequent MRI demonstrated persistent fluid in the subcutaneous fat, which tracked down to the level of the epidural space (Figure 2).

At this point, the neurosurgeon on call was consulted to evaluate the patient. The decision was made to return to the operating room once again for open repair of the CSF leak and lumbar drain placement. The procedure went without complication, and CSF was noted to be flowing from the lumbar drain at approximately 20-25 mL per hour. On postoperative day 2, the lumbar drain was observed to be yielding no fluid and was thus removed. However, shortly after removal, the patient was found to have persistent CSF drainage, this time from his midline incision. Due to his refractory CSF leakage, the neurosurgeon and neurointensivists had an extensive discussion with the patient and his mother regarding optimal postoperative care. Up to this point, the patient reported difficulty maintaining bed rest in the supine position due to his severe pain. As a result, he had been frequently adjusting his position and sitting up, which likely contributed to the difficulty in treating his CSF leak.

After a thorough discussion with the patient and his mother, the team opted to return to the operating room and place two lumbar drains, utilizing a primary drain but placing the second and clamping it so that it could be used as a backup should the other drain fail. With patient consent, the difficult decision was also made to keep the patient intubated postoperatively and to place him in a Roto bed, confined to the prone and lateral positions, to allow his incisions to heal without pressure, in hopes that the CSF leak would definitively heal. The patient continued to drain an average of 20 mL per hour through his lumbar drain. On postoperative day 11, the drains were removed, and an epidural blood patch (EBP) was performed at the sites of the lumbar drain entry sites in an attempt to prevent CSF leakage from the lumbar drain dural entry points. On postoperative day 13, the patient was extubated and reported no headaches or lower extremity pain. The patient was observed for several more days, and there was no evidence of continued CSF leakage. At this time, the patient was cleared for discharge from a neurology and neurosurgery perspective.

## Discussion

Dural tears are the most common complication in spine surgery, with reported incidence ranging anywhere from 0.5% to 18% [1,5,6]. However, it is believed that spine surgeons underestimate the frequency of this complication [7]. The risk factors for dural tears include advanced age, anatomical irregularities, revision surgery, thinning of the dura, and surgeon inexperience [6,8]. Dural tears are more common in patients who underwent a previous surgery due to the development of scar tissue, altered anatomy, inadequate dissection planes, and the adherence of surrounding tissues to the dura. Minimally invasive spinal surgery, with small incisions and a muscle-splitting approach, can be used to decrease the likelihood of dural tearing, thereby decreasing the chance of persistent CSF leakage as well as the amount of dead space created [7,9]. The most challenging situation after a persistent postoperative CSF leak is an unrecognized durotomy. In such cases, the diversion of CSF using a closed subarachnoid catheter is widely used with reported success rates between 85% and 94% [10]. However, the complication rate is excessively high, with some reports estimating up to 44.4%, and the most severe complications include over-draining, pneumocephalus, and meningitis [10].

While published literature regarding the incidence of dural tears with minimally invasive spinal surgery exists, the types of dural tears that require closure remain poorly understood. According to the limited amount of existing literature available, recommendations for the treatment of dural tears include primary repair, closed subarachnoid drainage through a subarachnoid drainage catheter, grafting (which may consist of muscle, fat, fascia, fibrin adhesive, blood patches, or cyanoacrylate polymer sealant), application of Gelfoam® to the tear, and bed rest [10-12]. However, there are no established treatment guidelines between the different etiologies of CSF leakage. The most important aspect in the management of CSF leakage is prevention, including preoperative risk assessment and meticulous intraoperative manipulation. One such example includes addressing iatrogenic residual bone spikes from bone drilling or uneven bites with a rongeur. Any movement that increases intrathecal pressure (e.g., violent awakening from anesthesia, coughing, and straining during defecation or urination) could potentially create a dural tear in a patient with residual bone spikes. When undergoing revision surgery, it is recommended to begin the dissection in areas without scar tissue and carefully proceed toward the more scarred regions [11,13].

One of the most widely accepted therapies for dural tearing is direct suture repair, although many reports estimate a failure rate of 5%-9% [3]. One treatment strategy that has been proposed is to alter the pressure gradient in a manner that stops CSF leakage. This method is based on one of two mechanisms: either reducing the subarachnoid fluid pressure or increasing the epidural space pressure [4,14] in addition to direct suture or augmented closure with dural substitute material [3]. It has been reported that unrecognized dural tearing during surgery occurs in approximately 6.8% of cases with a rate of spontaneous CSF leak cessation around 80%-95% [3]. The flow of CSF in the subarachnoid space is based on pressure gradients between any two communicating points [4,15]. Therefore, CSF leakage could theoretically be slowed by decreasing the existing pressure gradients, which can be done by reducing the subarachnoid fluid pressure or increasing the epidural space pressure. The methods to reduce subarachnoid fluid pressure include inhibiting the production of CSF, prolonged recumbent positioning, and CSF shunting via subarachnoid catheterization.

The other method involving the alteration of pressure gradients to stop CSF leakage is increasing the epidural fluid pressure. An epidural CSF seroma may develop secondary to a dural tear, which can eventually seep into the tissue surrounding the incision. When the CSF outflow exceeds the strength of the sutured tissue, it will create communication between the intradural space and the external wound. Therefore, a tight fascial closure, which increases the epidural fluid pressure, thereby stopping the CSF flow and facilitating adherence of the dural flaps, is required to avoid CSF leakage. While awaiting healing of the dural tear, a subfascial drain can be placed to clear excess CSF and thereby eliminate dead space formation, allowing the fascia to heal, thereby preventing CSF leak from the incision [15]. One other way in which some physicians have increased the epidural fluid pressure is through epidural blood patches (EBPs). EBPs not only help seal the CSF leak but also increase the epidural fluid pressure. As a result, in patients with an uncertain site of CSF leakage, EBPs can be extremely valuable [16]. Each of the aforementioned methods was attempted in the presented case. However, none were ultimately successful until the patient was sedated and intubated to allow for appropriate postoperative activity restrictions.

Regarding this case, conservative measures ultimately resulted in the cessation of the CSF leak. Prolonged bed rest is a useful adjunct in the treatment of CSF leaks, and patient positioning to control intracranial pressure is imperative. The lumbar subarachnoid CSF pressure is highest in the sitting and standing positions. However, the subarachnoid pressure is lower in the supine position with foot elevation. Given the overall patient comfort in this position in conjunction with its efficacy, the supine position is commonly used in the treatment of CSF leaks. However, the patient had difficulty maintaining this position, which may have negatively impacted his treatment course. Therefore, we opted to utilize prone and lateral positioning. The subarachnoid CSF pressure is lowest in the prone position. To maintain patient positioning, sedation and intubation were used [17].

## Conclusions

In the present report, we have described the challenging case of a persistent, refractory postsurgical spinal CSF leak, as well as our management. Most patients with postoperative CSF leaks experience spinal-type headaches, which prevent them from laying in the supine position for extended periods of time. Most patients are able to lay supine in a bed for 1-2 weeks, allowing for appropriate repair, but our patient was repeatedly found sitting and standing with a persistent CSF leak. The decision was made to keep the patient intubated and lateral/prone to allow for continuous monitoring of the dressing for CSF leak as well as optimal reduction of pressure on the operative site. Thus, understanding suitable positioning techniques to reduce leakage is crucial for proper management.
